# Effect of Relaxation Properties on the Bonding Durability of Polyisobutylene Pressure-Sensitive Adhesives

**DOI:** 10.3390/polym17172297

**Published:** 2025-08-25

**Authors:** Anna V. Vlasova, Nina M. Smirnova, Viktoria Y. Melekhina, Sergey V. Antonov, Sergey O. Ilyin

**Affiliations:** A.V. Topchiev Institute of Petrochemical Synthesis, Russian Academy of Sciences, 29 Leninsky Prospect, 119991 Moscow, Russia

**Keywords:** pressure-sensitive adhesives, polyisobutylene, contact time, durability, relaxation, adhesion, rheology

## Abstract

Pressure-sensitive adhesion arises at a specific rheological behavior of polymer systems, which should correlate with their relaxation properties, making them potentially useful for predicting and altering adhesive performance. This work systematically studied the rheology of eco-friendly pressure-sensitive adhesives based on non-crosslinked polyisobutylene ternary blends free of solvents and byproducts, which serve for reversible adhesive bonding. The ratio between individual polymer components differing in molecular weight affected the rheological, relaxation, and adhesion properties of the constituted adhesive blends, allowing for their tuning. The viscosity and viscoelasticity of the adhesives were studied using rotational rheometry, while their adhesive bonds with steel were examined by probe tack and shear lap tests at different temperatures. The adhesive bond durability at shear and pull-off detachments depended on the adhesive composition, temperature, and contact time under pressure. The double differentiation of the continuous relaxation spectra of the adhesives enabled the accurate determination of their characteristic relaxation times, which controlled the durability of the adhesive bonds. A universal linear correlation between the reduced failure time of adhesive bonds and their reduced formation time enabled the prediction of their durability with high precision (Pearson correlation coefficient = 0.958, *p*-value < 0.001) over at least a four-order-of-magnitude time range. The reduction in the formation/failure times of adhesive bonds was most accurately achieved using the longest relaxation time of the adhesives, associated with their highest-molecular-weight polyisobutylene component. Thus, the highest-molecular-weight polymer played a dominant role in adhesive performance, determining both the stress relaxation during the formation of adhesive bonds and their durability under applied load. In turn, this finding enables the prediction and improvement of adhesive bond durability by increasing the bond formation time (a durability rise by up to 10–100 times) and extending the adhesive’s longest relaxation time through elevating the molecular weight or proportion of its highest-molecular-weight component (a durability rise by 100–350%).

## 1. Introduction

Pressure-sensitive adhesives (PSAs) have been known for a long time, finding applications in medicine [1,2,3,4], transdermal drug delivery [5,6,7], different technologies [8,9,10,11,12], and everyday life [13]. PSAs are polymers or their mixtures that form adhesive bonds upon short-term contact at room temperature [14]. Non-crosslinked PSAs frequently outperform crosslinked ones thanks to their eco-friendly nature, which results from the absence of chemical crosslinking reagents or solvents and reusability [15,16,17]. PSAs engage three factors to form an adhesive bond: tack, cohesion, and adhesion [18,19]. Tack is necessary when an adhesive starts contacting a substrate. Cohesion is an internal holding power of an adhesive, i.e., a degree to which an adhesive itself retains internal integrity. Adhesion is a strength of a bond created between an adhesive and a substrate surface [20,21,22]. During the formation of an adhesive bond under external forces, internal stresses accumulate within the adhesive adjoining the interface, which may reduce the resulting strength of the adhesive bond. The internal stresses can relax, but it requires a certain amount of time [23]. However, an acceleration of the stress relaxation is also possible for potentially strengthening an adhesive bond by exposing it to elevated temperatures [24]. In turn, the investigation of the rheological properties of adhesives can determine their relaxation properties, which are associated with the durability of adhesive bonds [25].

There are many polymers suitable for producing PSAs, including acrylic polymers [26,27], silicones [21,28], polyurethanes [29,30], epoxy resins [31,32], natural rubber [33,34], different biopolymers [35,36], and various hybrid blends of these materials [37]. Nevertheless, non-crosslinked PSAs are mainly based on styrene block copolymers [38,39,40] and polyisobutylene (PIB) [41,42]. PIB is easier to synthesize and does not require plasticization with miscible hydrocarbon resins to acquire the essential characteristic of PSAs—a tackiness at applied load. However, to achieve the set of necessary rheo-adhesion properties, including the tackiness and the maintenance of an adhesive bond after removing the applied load, PIB must have a broad molecular weight distribution [43]. A necessary high macromolecular dispersity materializes usually by mixing polyisobutylenes with different molecular weights [44,45]. The low-molecular-weight components provide tack, while the high-molecular-weight components give the adhesive bond its strength [46].

**Figure 1:**
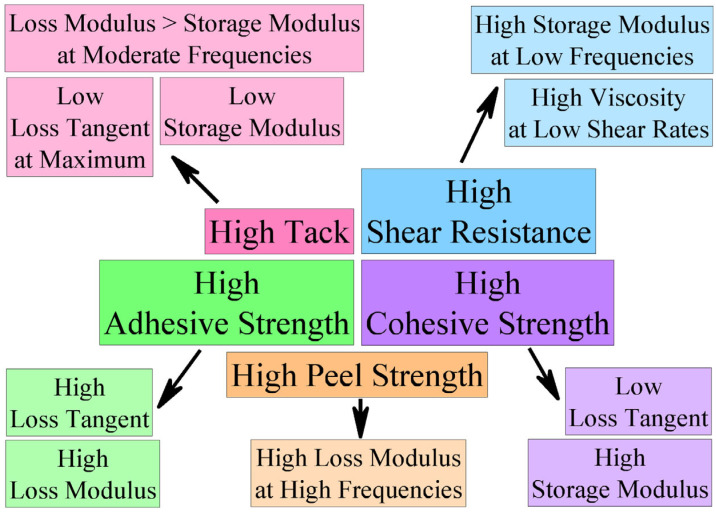
A general scheme of the relationship between adhesive characteristics and viscoelasticity of pressure-sensitive adhesives (adapted from [55]).

Tack, shear durability, and peel/pull-off strength usually describe the mechanical properties of PSAs [47,48,49], which strongly depend on their viscoelastic properties [50,51,52,53,54]. Moreover, the improvement of some adhesive properties requires a change in viscoelastic behavior, which simultaneously leads to deterioration in other adhesive characteristics (Figure 1). For example, high adhesive strength demands a high loss tangent [55]. In contrast, high cohesive strength occurs at a low loss tangent. A low storage modulus promotes high tack, but a high storage modulus is required to achieve strong cohesion strength and shear resistance. Thus, the relationship between apparent adhesion performance and viscoelasticity of a PSA is complex, as is the interplay among individual adhesive parameters. Earlier, the durability of adhesive bonds formed by low-viscosity non-crosslinked polyisobutylene-based PSAs was linked with their rheological properties [43]. However, the found correlation between the shear resistance and the complex modulus has significant limitations in practical application, as noticeable discrepancies arise between the calculated and experimental values of adhesive strength for high-molecular-weight or chemical crosslinked PSAs. The applicability of the strength/modulus correlation is fundamentally limited to cases of dominant shear loads acting on adhesive bonds.

Another more general approach to linking the durability of adhesive bonds, the conditions of their formation, and the relaxation characteristics of adhesives was to apply the reduced generalized curves [56]. The dependencies of the reduced durability versus the reduced contact time presented a universal description of the adhesive properties, where their reduction consisted of dividing by the relaxation time of a PSA. However, this correlation was correct only for one adhesive system, based on a polyvinylpyrrolidone/polyethylene glycol mixture with a specific component ratio, and at a single temperature. This work explores this approach using various compositions of adhesive mixtures at different temperatures of testing adhesive bonds.

However, removable PSAs must exhibit a certain level of adhesive strength—neither too low nor too high—to ensure sticking and then to avoid damaging the surface upon PSA removal. Typically, removable and semi-removable PSAs have peel strengths in the ranges of 2–4 and 6–8 N/cm, respectively [57]. Thus, the goal is not to develop removable PSAs with high adhesive strength, as this would compromise their intended function and effectively turn them into permanent PSAs. Instead, the key challenge lies in reliably predicting adhesive performance based on fundamental material properties. The significance of this study lies in establishing a universal correlation between the rheological, relaxation, and adhesive properties of pressure-sensitive adhesives. The work aims to investigate, for the first time, the relationship between the rheo-relaxation characteristics of non-crosslinked PSAs based on polyisobutylene blends and the durability of the adhesive bonds they form. It demonstrates how high-performance removable PSAs can be formulated through simple blending of readily available commercial polymers, thereby enabling a transition toward eco-friendly adhesives by eliminating the need for solvents, emulsifiers, crosslinkers, UV curing, and high-temperature bonding processes. This approach combines sustainability, simplicity, and predictability—offering a practical and environmentally responsible pathway for adhesive design.

## 2. Materials and Methods

### 2.1. Materials

The adhesive formulations PIB1–PIB4 used in this work were miscible blends of low-molecular-weight polybutene (INEOS Oligomers, League City, TX, USA) with two polyisobutylenes (BASF, Ludwigshafen, Germany) having different molecular weights. Table 1 presents the molecular weights of these polymers by gel permeation chromatography and the compositions of the resultant adhesives. PIB1 was previously used as a base adhesive for preparing composite PSAs, as it inherently tends to creep or cold flow [58]. The other formulations were variations of PIB1, designed with the practical aim of enhancing creep resistance without filling with solid particles. PIB2 and PIB3 reflect compositions with increased content of the highest-molecular-weight component B100, which may enhance the durability of adhesive bonds by improving resistance to permanent shear. An alternative approach was adopted in PIB4, which contains a reduced concentration of the lowest-molecular-weight component H1900. This modification was also expected to reduce cold flow and improve shear resistance by minimizing the fraction of highly mobile, low-viscosity chains. These compositional variations enable systematic tuning of viscoelastic and relaxation properties to achieve improved performance while maintaining the simplicity of a physically blended, solvent-free system.

The adhesive blends were prepared by melt mixing using a mixer Polydrive (Haake, Karlsruhe, Germany) equipped with sigma-blade rotors. First, Oppanol B100 and Oppanol B12 were mixed in a weight ratio of 1/2 with a gradual decrease in temperature from 180 °C to 80 °C for 60 min. Then, the remaining amounts of Oppanol B12 and Indopol H1900 were added, and the mixing was performed at a rotor rotation speed of 10 rpm for at least 6 h at 80 °C. Thus, no synthesis was involved in the preparation of the adhesives under study. Moreover, the simple physical blending of their components meant the order and temperature of mixing did not affect the final properties of the resultant PSAs, which eliminated the need for a detailed preparation scheme. In turn, the avoidance of chemical synthesis excluded the formation of by-products, which reduced environmental impact, while also keeping material costs low by using the available commercial polymers. Furthermore, the simplicity of the mixing process enabled the production of these adhesives using inexpensive equipment by low-skilled personnel, further reducing resource and labor costs.

Adhesive films for relaxation and durability studies were formed between two layers of a siliconized anti-adhesive poly(ethylene terephthalate) film PPI 0501 (PPI Adhesive Products, Waterford, Ireland) on a laminator HLCL-1000 (Cheminstruments, Fairfield, OH, USA) at 50 °C. The thickness of the resultant adhesive films was 150 ± 10 μm.

### 2.2. Methods

The rheological properties of the adhesive mixtures were studied on a rotational rheometer DHR-2 (TA Instruments, New Castle, DE, USA) at 25, 60, 100, and 140 °C using parallel plates with a diameter of 8 mm and an interplate distance of 0.5 mm. The flow curves were obtained via a stepwise increase in the shear rate from 0.01 to 100 s^−1^. Frequency dependencies of the storage and loss moduli were measured in the linear viscoelasticity region at a strain of 0.1% and a variation in the angular frequency from 0.628 to 628 rad/s.

**Figure 2:**
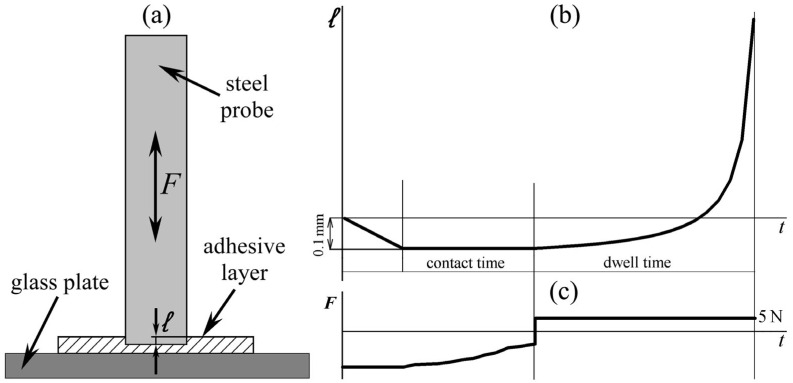
A scheme of probe tack tests (**a**) and profiles of the immersion depth (**b**) and force (**c**) during the tests.

The durability of adhesive bonds was investigated using the probe tack method on a texture analyzer TA.XT+ (Stable Micro Systems, Godalming, UK) at 25, 40, and 60 °C. An adhesive film under test was transferred onto a glass plate (Figure 2a), and a steel cylindrical probe with a diameter of 9.5 mm was immersed into it at a speed of 0.1 mm/min to a depth of 100 μm. Then, the contact with the probe was maintained for 5, 60, or 300 s, and the probe was torn off under a constant force of 5 N, recording the time for complete failure of the adhesive bond. Figure 2b,c show the evolution of the penetration depth of the probe into the adhesive and the force applied to the probe (first directed toward the adhesive and then away from it) during the test, respectively. The steel probe has an average arithmetic deviation of the surface roughness profile of 20.5 nm according with the ISO 25178 standard [59]. The measurements of time to failure were repeated at least 3 times.

**Figure 3:**
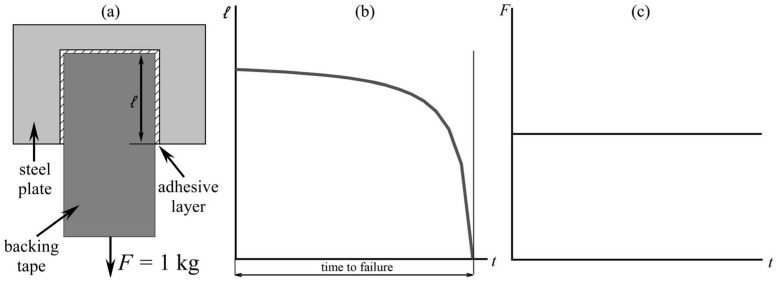
A scheme of shear load tests (**a**) and profiles of the glued length (**b**) and force (**c**) during the tests.

In addition, the durability of adhesive bonds at static shear load was studied using an S-RT-10 device (Cheminstruments, Fairfield, OH, USA) according to Procedure A of the ASTM D 3654 standard [60]. An adhesive sample with a width of 25.4 mm was applied to a commercial adhesive tape on one side and to a polished steel plate on the other side, and then rolled twice with a standard 2 kg roller. Before testing, the prepared tape/adhesive/steel joints were kept in a heating oven at 25, 40, or 60 °C for 25 min. After removal from the heating oven, the adhesive joints were allowed to cool for 5 min at 25 °C. Then, they were fixed vertically via their steel sides and loaded via their tape sides with standard loads of 1 kg (Figure 3a). The time until the failure of the adhesive joint was recorded while repeating the experiment at least 3 times. Figure 3b,c illustrate the evolution of the length of the adhered part of the backing tape and the force applied to it during the test, respectively.

## 3. Results and Discussion

### 3.1. Rheology of Adhesives

The PSA samples under study were physical miscible blends in which no chemical or specific physicochemical interactions were present. Their structure and IR spectra were similar to those of any polyisobutylene. Nevertheless, the different molecular weights of the PSA-constituent polymers and their varying ratios in the PSA composition enabled the alteration of the rheological and adhesive properties of the resultant blends.

**Figure 4:**
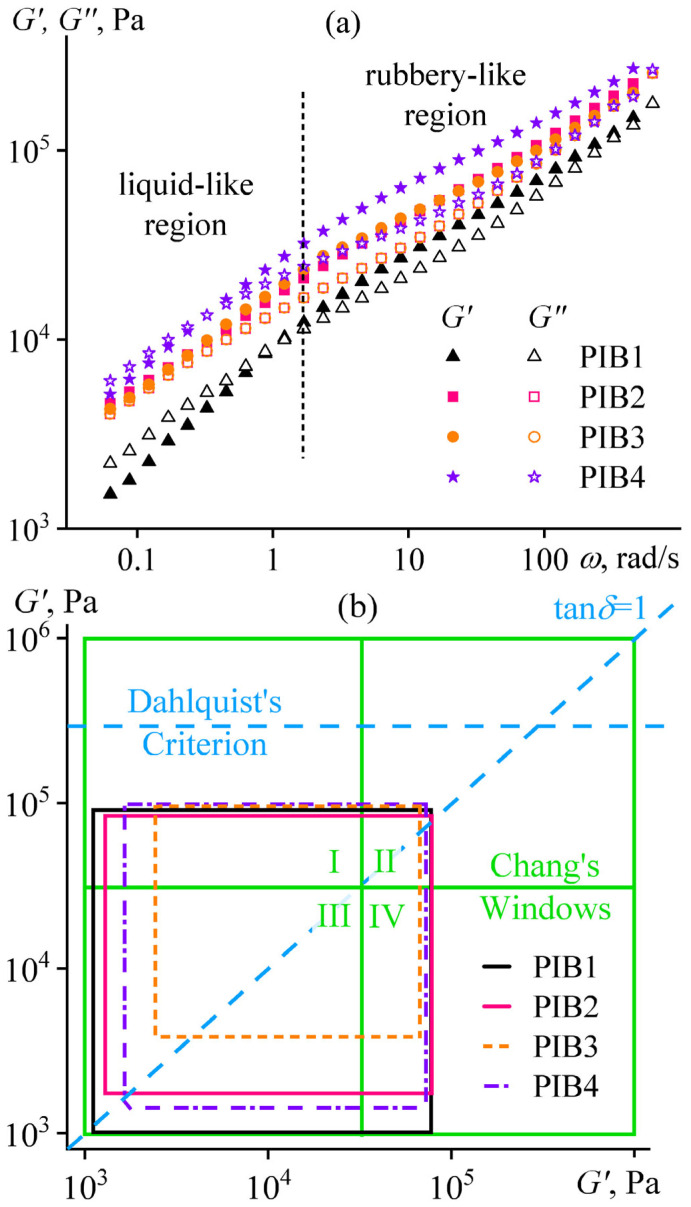
Frequency dependencies of the storage moduli *G*′ and loss moduli *G*″ (**a**) and Chang’s viscoelastic windows (**b**) for the PIB1–PIB4 adhesive formulations at 25 °C.

For all adhesive formulations, the storage and loss moduli were comparable and grew with an increase in the angular frequency (Figure 4a). Two relaxation regions were distinguishable, but their transition was very smooth because of the high dispersity of the macromolecules in the adhesives: the liquid-like and rubbery-like states at low and high frequencies, respectively. A decrease in the ratio between the moderate and high molecular weight components (Oppanol B12 and B100) elevated the storage and loss moduli, but within certain limits. Thus, an increase in the B100 content from 10% to 15% raised the storage modulus by 60% (at *ω* = 3 rad/s), while a further increase to 20% elevated the modulus by only 9%.

A reduction in the proportion of the low-molecular-weight component (Indopol H1900) also allowed for achieving higher storage and loss moduli. A twofold decrease in its content in the adhesive composition from 40% to 20% increased the storage modulus by 2.5 times. In all cases, these limited variations of the adhesive composition also enabled changing the ratio between the storage and loss moduli, in addition to their absolute values, i.e., altering the proportion of reversible and permanent deformations.

A convenient way to evaluate the quality and intended application of a PSA is to use the viscoelastic windows proposed by Chang [61]. The relationship between the storage and loss moduli of an adhesive is represented as a square, where the bottom-left corner corresponds to the dynamic moduli measured at *ω* = 0.01 rad/s and the top-right corner corresponds to the moduli at 100 rad/s. For a polymeric material to function as a PSA, its square must fall within the Chang viscoelastic window, which spans from 10^3^ to 10^6^ Pa for both *G*′ and *G*″. This window is further divided into four quadrants:

- Quadrant I (top-left, high *G*′, low *G*″) corresponds to anti-adhesive coatings;
- Quadrant II (top-right, high *G*′ and *G*″) describes PSAs with high resistance to shear loads;
- Quadrant III (bottom-left, low *G*′ and *G*″) represents removable and medical adhesives;
- Quadrant IV (bottom-right, low *G*′, high *G*″) points on PSAs with fast adhesion at low temperatures.

In our case, the squares for all studied PSAs lie within Quadrant III (Figure 4b), which is consistent with their intended use for reversible adhesive bonding. In addition, the storage modulus of the adhesives is below 3·10^5^ Pa, satisfying the Dahlquist criterion for good tack in PSAs [62,63]. Furthermore, the bottom-left and top-right corners of the adhesive squares intersect the line corresponding to a loss tangent (tan*δ* = *G*″/*G*′) of 1.0, where energy storage during deformation equals its dissipation. This fact indicates an ideal balance between elasticity (providing an adhesive failure) and viscosity (providing a cohesive failure), promoting maximization of the apparent adhesive strength. Indeed, the adhesive strength of all PIB-based PSAs is comparable—7.1 ± 0.4 N/cm at 90° peel from the steel surface under a rate of 30 cm/min at 25 °C, confirming their optimized performance and semi-removable character [56].

**Figure 5:**
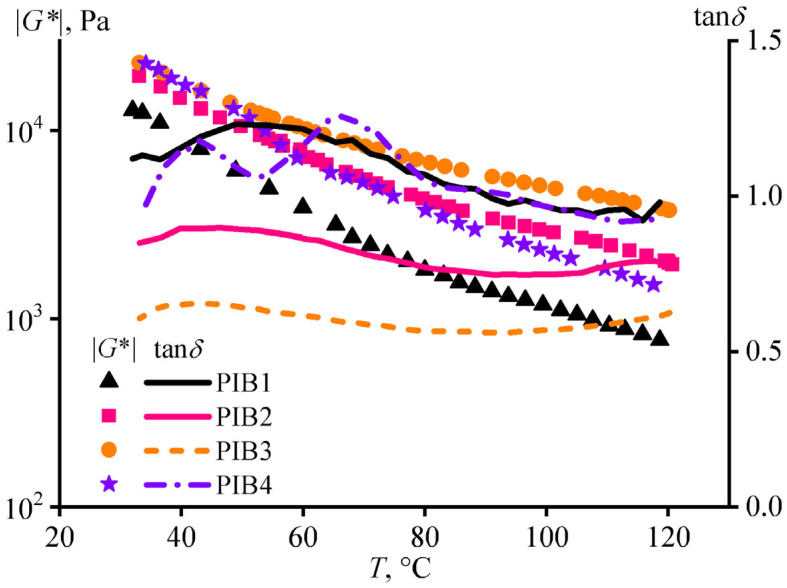
Temperature dependencies of the complex modulus and loss tangent for the PIB1–PIB4 adhesive formulations at the angular frequency of 1 rad/s.

PSAs should retain their rheological and adhesion properties at elevated temperatures to maintain formed adhesive joints under varying daytime temperatures or exposure to sunlight. Dynamic tests under smooth temperature rise allowed for estimating the evolution of the adhesives’ rheological properties (Figure 5). Regardless of temperature, the ratio between the storage and loss moduli, expressed by the loss tangent, remained almost constant for all formulations. Thus, the adhesives had stable viscoelastic properties over a wide temperature range. In other words, a sample that exhibits predominant elasticity and creep resistance will not become a liquid upon a temperature rise [64,65].

However, an increase in temperature reduced the absolute values of the storage and loss moduli because of a decrease in the complex modulus (Figure 5). Under high temperatures, the adhesives will be able to withstand less applied stress and for a shorter time. Nevertheless, an adjustment of adhesive composition enabled leveling out this rheology deterioration upon heating, scilicet by increasing the proportion of the high-molecular-weight polymer. For example, the formulations PIB2, PIB3, and PIB4 had close complex moduli at low temperatures. However, a temperature rise decreased the complex modulus more for adhesives that had smaller B100 content (see Table 1). In addition, adhesives with a higher B100 content also had a lower loss tangent, behaving more like solids.

**Figure 6:**
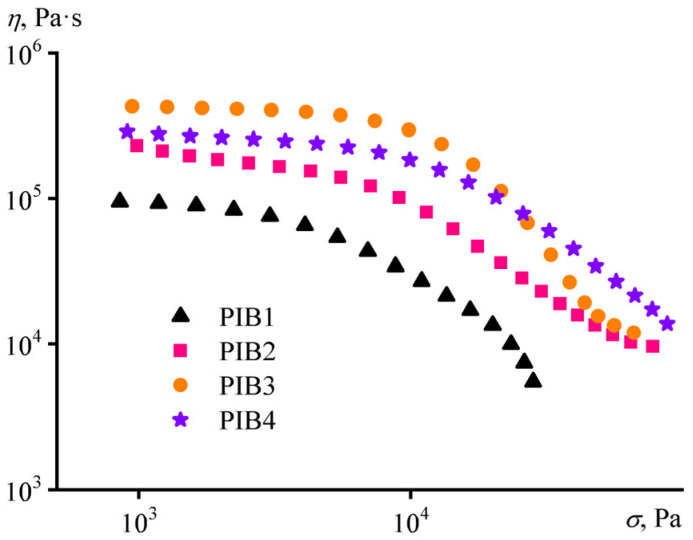
Dependencies of viscosity on shear stress for the PIB1–PIB4 adhesive formulations at 25 °C.

Flow curves of adhesives can assess both their behavior under shear testing and their ability to spread over a surface under applied compression (Figure 6). At low shear stresses, the viscosity of the samples was practically constant, being the factor that governs their shear resistance. In the region of high shear stresses, the viscosity decreased with an increase in the applied stress. This region is responsible for the formation and quality of the adhesive bonds with the substrate, e.g., for filling in the relief unevenness on a rough steel surface. An increase in the content of high-molecular-weight components elevated the viscosity of the adhesives, as was observed with a rise in their storage and loss moduli. However, there was also a difference: when the B100 proportion increased from 15% to 20% (PIB2 turned into PIB3), the low-shear viscosity of the samples continued to rise significantly, by a factor of 2.5, in contrast to their moduli (Figure 4). The explanation lies in the fact that the value of the rubbery plateau modulus of a polymer is practically independent of its molecular weight, unlike low-shear viscosity. Nevertheless, an increase in the shear rate brought the viscosities of PIB2 and PIB3 together, as a result of their forced transition into the rubbery state. Thus, a variation of the component ratio allows for selecting adhesive compositions that provide the highest possible viscosity at low shear stresses to ensure the best shear resistance while maintaining relatively low viscosity at high shear stresses for forming a good-quality adhesive bond.

### 3.2. Relaxation of Adhesives

**Figure 7:**
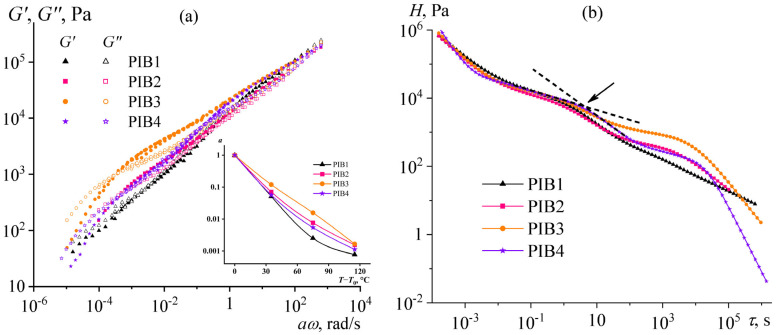
Frequency dependencies of storage and loss moduli for the PIB1–PIB4 adhesive formulations tested at various temperatures after their shift to 25 °C (**a**) and continuous spectra of relaxation times calculated from these data (**b**). The inset shows the dependencies of the shift factor on temperature. The arrow indicates an example of a hidden maximum on the relaxation spectrum and two tangents (shown by dashed lines) to its vertex.

In addition to rheological characteristics, the relaxation properties of adhesives are influential, scilicet their relaxation times at low observation times of adhesive bond formation and long observation times for adhesive bond destruction under static load. To determine the relaxation times, one can use the frequency dependencies of the storage and loss moduli measured at different temperatures and normalized to a single temperature for a wide coverage of possible relaxation times. According to the principle of time–temperature superposition and the Williams–Landel–Ferry (WLF) equation [66]:

(1) $$loga=log\frac{G^{\prime \prime }}{{G^{\prime \prime }}_{0}}=log\frac{G^{\prime }}{G_{0}^{\prime }}=\frac{-c_{1}\left(T-T_{0}\right)}{c_{2}+\left(T-T_{0}\right)},$$

where *G*′_0_ and *G*″_0_ are the storage and loss moduli at the reference temperature *T*_0_, *c*_1_ and *c*_2_ are the fitting parameters, and *a* is the shift factor, which shows by how much the frequency dependencies of the moduli shift along the frequency axis when the test temperature changes. In our case, all samples followed the WLF equation, and the frequency dependencies of the dynamic moduli at 25, 60, 100, and 140 °C enabled the obtaining of a broad frequency sweep of the moduli by their reduction to 25 °C (Figure 7a). The obtained master curves allow the projection of the adhesives’ behavior under a load applied over long test times, which is inversely proportional to the angular frequency: *t* = 1/*ω*. For example, the lowest reduced angular frequency of 10^−5^ rad/s corresponds to an observation time of 10^5^ s or 28 h. At these observation times, the PIB3 containing the highest B100 mass fraction exhibited the most elevated stiffness, which should provide the highest durability to its adhesive bonds.

However, the ability to form an adhesive bond at short loading times of about 0.1–1 s (an exposure rate of 1–10 s^−1^) is also critical. According to the Dahlquist criterion, the storage modulus of a PSA under these conditions must be less than 3 × 10^5^ Pa to form a strong adhesive bond [67,68]. In our case, all adhesive formulations met this condition, and the increased content of B100 in PIB3 should not interfere with the formation of an adhesive bond. Interestingly, an increase in the B100 content also resulted in a less pronounced temperature dependence of the rheological properties, as the shift factor for PIB3 was the least temperature-dependent (inset of Figure 7a). In other words, PIB3 should better maintain the stability of adhesive properties under temperature alterations. In contrast, the shift factor of PIB1 changed most strongly upon a temperature rise, indicating its higher sensitivity to the test temperature.

The obtained frequency dependencies or master curves enabled the calculation of continuous spectra of relaxation times for the adhesives under investigation [69,70]:

(2) $$G^{\prime }\left(\omega \right)=\int_{-\infty }^{\infty }H\left[\ln\left(\tau \right)\right]\left(\frac{\omega^{2}\tau^{2}}{1+\omega^{2}\tau^{2}}\right)d\left(ln\tau \right),$$

(3) $$G\prime \prime \left(\omega \right)=\int_{-\infty }^{\infty }H\left[\ln\left(\tau \right)\right]\left(\frac{\omega \tau }{1+\omega \tau }\right)d\left(ln\tau \right),$$

where $\;H\left[\ln\left(\tau \right)\right]$ and *τ* are the spectral strength and relaxation time, respectively. In our case, the spectral intensity for all samples decreased smoothly when the transition to the long-time region occurred (Figure 7b). However, all spectra contained one or two shoulders, or hidden maxima, as indicated by an arrow in Figure 7b. The presence of these shoulders may be due to the three types of polymers included in the adhesive compositions, since they differed significantly in molecular weight (Table 1). As a result, the medium- and high-molecular-weight components of the adhesives (B12 and B100, respectively) had longer relaxation times manifested as long-term shoulders in the relaxation spectra. For example, PIB3 had the highest content of the highest-molecular-weight B100, which echoed in a pronounced shoulder at a relaxation time region of 10^3^–10^4^ s (Figure 7b).

**Figure 8:**
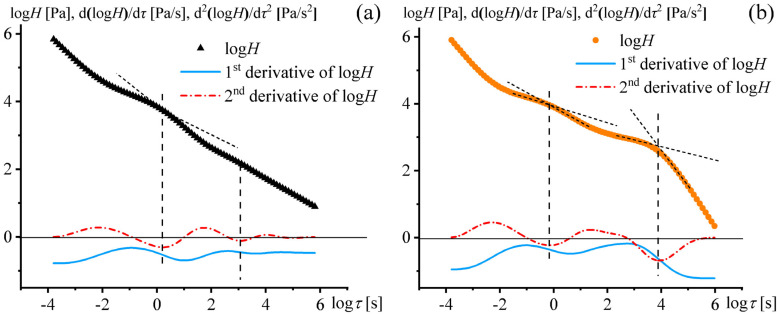
Dependencies of spectral strength on relaxation time and their 1st and 2nd derivatives for PIB1 (**a**) and PIB3 (**b**). The short dashed lines indicate tangents to the hidden maxima, while the vertical dashed lines mark the positions of their vertices.

The position of the relaxation times of the adhesives can be more accurately identified using the first and second derivatives of the logarithm of the spectral strength by the logarithm of the relaxation time: dlog*H*/dlog*τ* and d^2^log*H*/dlog*τ*^2^, respectively (Figure 8). The differentiation caused the hidden maxima to appear on the first derivative as inflection points of the decreasing curve. The second derivative allowed for identifying the hidden maxima more clearly by expressing them as local minima. Moreover, the second derivative enabled the visualization of undetected relaxation maxima, as in the case of the second long-time maximum of PIB1 around log*τ* = 3 (Figure 8a).

Thus, all PIB1–PIB4 adhesives had two hidden relaxation time maxima, which coincide with the minima of the 2nd-order derivatives. The ease of locating the minima’s positions allowed for determining the characteristic relaxation times given in Table 2. The first and second relaxation times differed from each other in a ratio *τ*_2_/*τ*_1_ by a factor of 780–10,600. In turn, the molecular weights of polyisobutylenes B12 and B100, which made up the adhesives and determined these relaxation times, correlated with each other as *M*_w,B12_/*M*_w,B100_ = 1/22. Since *τ* ~ *η* ~ *M*_w_^3.4^ [71], their relaxation times should differ in a ratio *τ*_B12_/*τ*_B100_ by a factor of 1/34,300, taking the degree exponent into account. The lower difference for the actual adhesive blends (*τ*_B12_/*τ*_B100_ ≈ *τ*_1_/*τ*_2_ = 1/780–1/10,600) may be due to the mutual solubility of the polymers, the decrease in their relative concentration (compared to 100%), and the presence of the low-molecular-weight plasticizing polybutene. The question is which of the two relaxation times (or both) affects the performance of the adhesives, giving them the greatest adhesive strength.

### 3.3. Adhesive Properties

**Figure 9:**
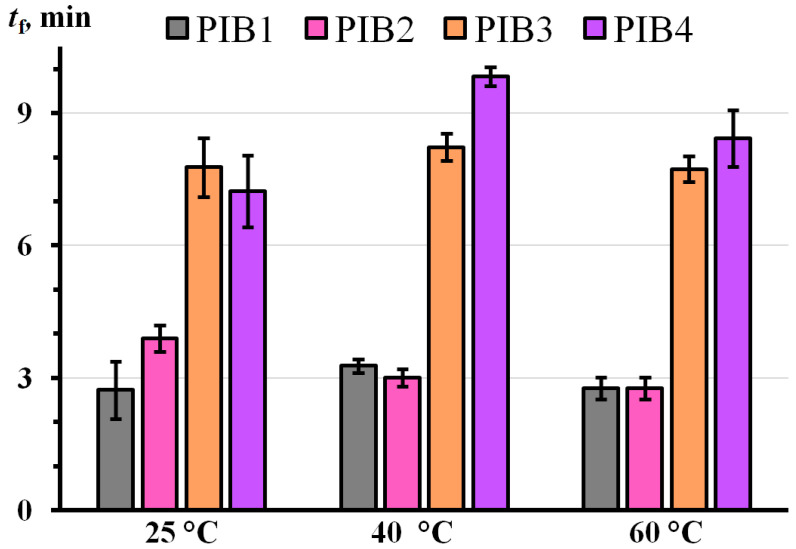
Durability of PIB1–PIB4 adhesive joints in shear bank tests at 25 °C. The abscissa axis indicates the temperatures at which the adhesive joints were pre-heated for 25 min before testing.

The durability of tape/adhesive/steel joints, as their time to failure (*t*_f_) under a shear load, was independent of the temperature and time of their preliminary holding in a heating oven (Figure 9). However, it depended on the adhesive composition. This result indicates that the relaxation of internal stresses created during bonding of the adhesive under external pressure occurred very quickly, even at 25 °C, i.e., it correlates with a fast relaxation time of 0.69–3.74 s (Table 2). In turn, the increase in the durability of adhesive joints when moving from PIB1 to PIB2 and then to PIB3 and PIB4 can result from the rise in the slow relaxation time from 1178 s to 21,880 s in this adhesive series. Thus, both approaches to improving shear resistance yield similarly positive results: increasing the proportion of the highest-molecular-weight component in PIB3 compared to the base formulation PIB1 (see Table 1) and decreasing the proportion of the lowest-molecular-weight component in PIB4. In both cases, the boost in durability is comparable, ranging from 150% to 200%, and is independent of temperature.

**Figure 10:**
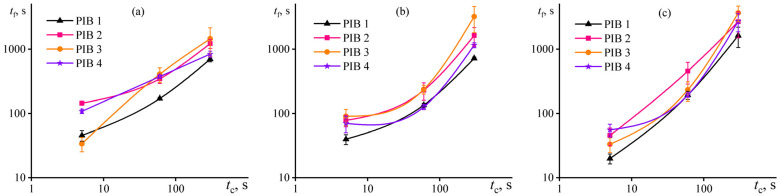
Durability of PIB1–PIB4 adhesion bonds in probe tack tests at 25 °C (**a**), 40 °C (**b**), and 60 °C (**c**) as a function of contact time under applied pressure.

The durability of adhesive/steel bonds was strongly dependent on the contact time (*t*_c_) during bond formation under applied pressure, as clearly shown by the results of stickiness probing at different temperatures (Figure 10). The longer the contact time, the longer the relaxation of internal stresses under pressure is, resulting in higher adhesion strength. In this case, a change in the contact time from 5 s to 5 min elevated the durability of adhesion bonds by one or two orders of magnitude. Meanwhile, a rise in the test temperature strengthened the effect of contact time on adhesive durability, which may result from a reduced relaxation time. On the one hand, the acceleration of relaxation lowers internal stresses more rapidly during holding of an adhesive under pressure, which should contribute to the growth of adhesive strength. Furthermore, an enhancement occurs in the flowability of the adhesive and, consequently, its ability to fill the roughness of the bonded surface. On the other hand, a decrease in relaxation time accompanies an acceleration of macromolecular dynamics, which may contribute to a more rapid failure of the adhesive bond due to the lower viscosity of the adhesive and its quicker ability to change shape by stretching under applied load. For example, a temperature rise reduced the adhesive strength of bonds formed under a contact time as low as 5 s but increased the adhesive strength under pre-pressure for 5 min. In addition, an increase in the proportion of the highest-molecular-weight component in the adhesive formulation (transition from PIB1 to PIB3) is generally more effective for enhancing the durability of adhesive bonds, particularly at long bond formation times. In this case, the improvement in durability ranges from approximately 100% to 350%, regardless of the test temperature.

**Figure 11:**
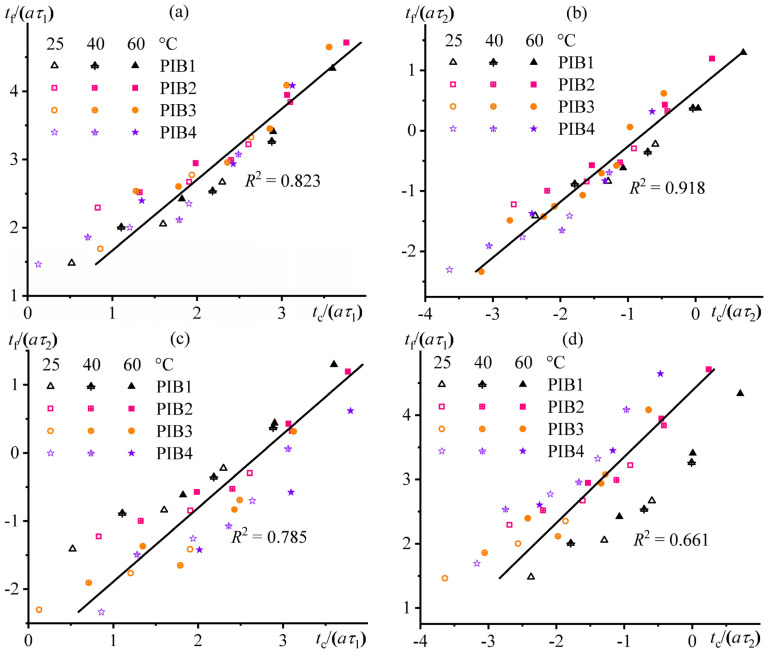
Correlation dependencies of the reduced time of adhesive bond failure on the reduced time of adhesive bond formation for polyisobutylene pressure-sensitive adhesives. The failure time was normalized by the fast (**a**,**d**) or slow (**b**,**c**) relaxation time, while the formation time was simultaneously normalized using the fast (**a**,**c**) or slow (**b**,**d**) relaxation time. The legends indicate the adhesive formulation and the test temperature used in the probe tack tests.

Thus, the two relaxation times and their variation with temperature have a complex effect on the adhesion strength. The correlations between the failure time (*t*_f_) and the contact time (*t*_c_) may help to establish which of the two relaxation times affected the adhesion performance to a greater extent. However, these correlations should be normalized using the fast and/or slow relaxation times (*τ*_1_ and *τ*_2_, respectively; see Table 2). In our case, the best correlation between *t*_f_ and *t*_c_ occurs after normalizing these times by the shear factor *a* (taking different test temperatures into account) and the long relaxation time *τ*_2_ (Figure 11b), demonstrating the highest determination coefficient (*R*^2^ = 0.918) and the highest Pearson linear correlation coefficient (*ρ*_P_ = 0.958, *p*-value < 0.001). Thus, the key to forming a strong adhesive bond is the relaxation of the highest-molecular-weight polymer B100, which is part of the adhesives, providing them with cohesive strength. Moreover, the relaxation rate is critical in both terms: for reducing internal stresses during the formation of adhesive bonds and for resisting stretching during their failure.

Interestingly, a good linear correlation also takes place when fast relaxation times *τ*_1_ are used for normalization (*R*^2^ = 0.823, *ρ*_P_ = 0.948, *p* < 0.001; Figure 11a). This effect is probably due to the influence of B12, which has the average molecular weight among the three polymeric constituents of these adhesives, on their final relaxation–adhesion properties. The use of heterogeneous relaxation times to find correlations increases the scattering of calculated points (Figure 11c,d). However, the scattering is slightly smaller in the case of comparing *t*_f_/*τ*_2_ and *t*_c_/*τ*_1_ than *t*_f_/*τ*_1_ and *t*_c_/*τ*_2_ (*R*^2^ = 0.785 versus 0.661, *ρ*_P_ = 0.886 versus 0.813, *p* < 0.001). This fact indirectly indicates that the adhesion bond formation time is more related to the fast relaxation time. In contrast, the failure time is more determined by the long relaxation time. Both results are entirely anticipated, given the duration of these processes.

Note that the presented correlations between the reduced times of adhesive bond formation and failure are independent of the choice of reference temperature, which provides them with a universal nature. In turn, the universality of the correlations allows predicting the durability of an adhesive bond at any temperature of interest, provided that its durability at another temperature and the temperature dependence of the relaxation time of a PSA are known.

## 4. Conclusions

A systematic study of the rheological, relaxation, and adhesion properties of non-crosslinked pressure-sensitive adhesives based on a polyisobutylene/polybutene mix at different temperatures and times of adhesive bond formation revealed the following:

- Double differentiation of a continuous relaxation time spectrum of an adhesive with respect to time in logarithmic coordinates enables the simple and accurate determination of its characteristic relaxation times, which are associated with the durability of its adhesive bonds.
- In a mixture of three polymers with different molecular weights, the higher-molecular-weight polymer plays a dominant role, determining both the stress relaxation during the formation of the adhesive bond and its durability under applied load.
- A way to improve adhesive bond durability is by extending both the bond formation time and the adhesive’s longest relaxation time. In turn, the relaxation time of the three-component adhesive elongates with an increase in (1) the proportion and (2) molecular weight of the highest-molecular-weight polymer, (3) a reduced content of the lowest-molecular-weight plasticizer, and (4) a lower operational temperature. In particular, the increased formation time of an adhesive bond enhances its durability by up to 10–100 times, whereas the greater proportion of the highest-molecular-weight polymer or the reduced amount of the lowest-molecular-weight component improves durability by 100–350%.
- There is a universal correlation between the time to failure of adhesive bonds and the time of their formation after dividing these times by the product of the relaxation time and the time–temperature shift factor, which respectively determine the relaxation rate and its dependence on temperature for an adhesive.
- The generalized dependence of the reduced durability on the reduced pressure-holding time enables the prediction of adhesive bond performance, thereby aiding in the development of new pressure-sensitive adhesives with improved adhesion characteristics.

This study is fundamental rather than applied in nature, providing a theoretical framework rather than novel adhesives with high specific characteristics. Nevertheless, it establishes quantifiable relationships between adhesives’ properties and performance. In turn, these insights lay the groundwork for rational design and optimization of adhesives in practical applications. However, the presented framework has limitations, as it applies only to pressure-sensitive adhesives whose adhesion to various substrates arises from physicochemical interactions rather than chemical bonding. Moreover, these adhesives must obey the time–temperature superposition principle, meaning they should be homogeneous blends, free from filler particles, dispersed droplets, a crystalline phase, or microphase separation, exhibiting a single dependence of relaxation time(s) on temperature. A specific limitation of the present study is its focus on a single adhesive system, specifically polyisobutylene blends. Future work should aim to extend the proposed approach to different pressure-sensitive adhesives (e.g., based on polydimethylsiloxanes, polyhexenes, polyvinylpyrrolidone/polyethylene glycol mixtures, or other miscible polymer blends) and to generalize the results from these diverse systems. These studies would help establish broader structure–property–performance relationships and enhance the predictive power and applicability of the framework.

## Figures and Tables

**Table 1:** Molecular weights of polymers and their mass fractions in pressure-sensitive adhesives.

Component	*M*_w_, kDa	*Đ* = *M*_w_/*M*_n_	PIB1	PIB2	PIB3	PIB4
Polybutene Indopol H1900, wt%	4.5	1.8	40	40	40	20
PIB Oppanol B12, wt%	51	3.2	50	45	40	66.7
PIB Oppanol B100, wt%	1100	4.4	10	15	20	13.3

**Table 2:** Relaxation times determined from the minima of the 2nd derivatives of the spectral strength logarithm with respect to the time logarithm for the PIB1–PIB4 adhesive formulations.

Sample	*τ*_1_, s	*τ*_2_, s
PIB1	1.51	1178
PIB2	0.74	2438
PIB3	0.69	7326
PIB4	3.74	21,880

## Data Availability

The raw data supporting the conclusions of this article will be made available by the authors on request.
